# Measuring the Candidates' Emotions in Political Debates Based on Facial Expression Recognition Techniques

**DOI:** 10.3389/fpsyg.2022.785453

**Published:** 2022-05-09

**Authors:** Alfredo Rodríguez-Fuertes, Julio Alard-Josemaría, Julio E. Sandubete

**Affiliations:** ^1^ESIC Business and Marketing School, Madrid, Spain; ^2^ESIC University, Madrid, Spain; ^3^Complutense University of Madrid, Madrid, Spain; ^4^CEU San Pablo University, Madrid, Spain

**Keywords:** neuromarketing techniques, facial expression analysis, emotions, election debates, political communication, iMotions platform

## Abstract

This article presents the analysis of the main Spanish political candidates for the elections to be held on April 2019. The analysis focuses on the Facial Expression Analysis (FEA), a technique widely used in neuromarketing research. It allows to identify the micro-expressions that are very brief, involuntary. They are signals of hidden emotions that cannot be controlled voluntarily. The video with the final interventions of every candidate has been post-processed using the classification algorithms given by the iMotions's AFFDEX platform. We have then analyzed these data. Firstly, we have identified and compare the basic emotions showed by each politician. Second, we have associated the basic emotions with specific moments of the candidate's speech, identifying the topics they address and relating them directly to the expressed emotion. Third, we have analyzed whether the differences shown by each candidate in every emotion are statistically significant. In this sense, we have applied the non-parametric chi-squared goodness-of-fit test. We have also considered the ANOVA analysis in order to test whether, on average, there are differences between the candidates. Finally, we have checked if there is consistency between the results provided by different surveys from the main media in Spain regarding the evaluation of the debate and those obtained in our empirical analysis. A predominance of negative emotions has been observed. Some inconsistencies were found between the emotion expressed in the facial expression and the verbal content of the message. Also, evidences got from statistical analysis confirm that the differences observed between the various candidates with respect to the basic emotions, on average, are statistically significant. In this sense, this article provides a methodological contribution to the analysis of the public figures' communication, which could help politicians to improve the effectiveness of their messages identifying and evaluating the intensity of the expressed emotions.

## 1. Introduction

It has been more than two decades since ideas related to the analysis about public communication of political leaders began appearing in the literature with a particular focus on parliamentary debates, public events and interviews, for a review see e.g., Shaw (2000), D'Errico and Poggi (2019), and Ortigueira-Sánchez and Cárdenas-Egúsquiza (2019). Politicians usually uses different strategies based on both verbal language (tone of speech, arguments, rhetorical figures, etc.), as well as non-verbal (gestures, looks, facial expressions, etc.) to increase the persuasive capacity of their messages (see Dumitrescu et al., 2015). Within the body language variables, facial expressions constitute the most basic communication elements of interpersonal communication. In this sense, Dumitrescu et al. (2015) point out that non-verbal communication is rich in content and that it has attracted significant attention from news coverage on television and in the campaigns of the candidates.

Many emotions that cannot be explained through words are transmitted through facial expressions. The ability to identify and evaluate these emotions can provide a lot of information about the person's condition and the communication strategies they follow specially to persuade voting publics (Elliott and Jacobs, 2013). D'Adamo et al. (2021) showed that the role played by emotions in audiovisual pieces of political communication, and therefore, in television debates within the framework of electoral campaigns, aims to generate a specific reaction in the voter. In this sense, the positivity or negativity that voters perceive and feel when exposed to the different candidates' communications is not random. On the contrary, the strategic planning of what emotions to convey in television debates and especially in the golden minute, is worked out during the pre-campaign phase, through field research, survey studies, analysis, and diagnostics.

These emotions, as asserted by Kahneman (2012), are present in the pieces of audiovisual political communication during the campaign period. Therefore, paying attention to these pieces is key to try to recognize the presence of positive and/or negative emotions, and their impact on the voter's subsequent decision and action. A triggering factor of these emotions, beyond the textuality of the message, the content of the discourse, is determined by non-verbal communication itself, the study of which in the framework of kinesics determines the importance of body posture, gesticulation, facial expression, and gaze (see Mancera, 2015). In this sense, as this author points out, the involvement of this discipline is direct in any communicative act. For this reason, it is called the primary discipline of non-verbal language (together with paralinguistics).

Despite the extensive literature referring to verbal and nonverbal communication in the political sphere, the study of these issues with the use of neuromarketing techniques is still a development field with important novel implications, for a review see e.g., D'Errico and Poggi (2019) and Dumitrescu et al. (2015). Particularly, we are going to focus on the application of the Facial Expression Analysis (FEA), a technique widely uses in neuromarketing research as stated by Fortunato et al. (2014), which allows the identification and evaluation of the intensity of the expressed emotions. In our case, this application is a methodological contribution to the analysis of the communication of public figures as politicians incorporating a new approach complementary to the other techniques commonly used in that field.

In this article, we provide the following contributions: First, we propose the application of novel facial expression techniques to identify the emotions that the main candidates participating in the general elections held in Spain on April 28, 2019. As far as we know, no papers have yet appeared in this regard. Mainly, we focus on some computer-based video classification algorithms because allow us to automatically encode the main facial expressions showed by the politicians as well as to identify the seven basic emotions (joy, anger, fear, surprise, disgust, sadness, and contempt).

Second, we associate the basic emotions with specific moments of the candidate's speech, identifying the topics they address and relating them directly to the expressed emotion. Third, we analyze whether the differences shown by each candidate in every emotion are statistically significant. In this sense, we apply the non-parametric chi-squared goodness-of-fit test. We also consider the ANOVA analysis in order to test whether, on average, there are differences between the candidates. Finally, we check if there is consistency between the results provided by different surveys from the main media in Spain regarding the evaluation of the debate and those obtained in our empirical analysis.

To sum up, the main interest of our paper is to illustrate that by choosing novel facial expression techniques to analyze the discourse of political leaders provides us with very interesting information when designing their communication policies, identifying the impact they can have on the electorate, the emotions they can transmit, and complements the rest of the classic analysis techniques in this context. The results obtained have been statistically validated and robust evidence has been obtained in this respect.

The article is organized as follows. Section 2 provides a literature review related to the role of emotions and facial expressions expressed by politicians in their communication strategies. Section 3 presents the theoretical framework that we have employed in this paper. Section 4 reports the main results of this article. Section 5 offers a discussion of the results obtained. Finally, Section 6 gives some concluding remarks.

## 2. Conceptual Framework

In this section we are going to provide a literature review about how to analyze the speech of political candidates during election debates from a neuromarketing point of view. The main objective of the communication made by political candidates during election campaigns is, on the one hand, to persuade potential voters, and on the other hand, to convey a coherent and solid image of the political option they represent (Dumitrescu et al., 2015). Both dimensions (verbal and non-verbal communication) play a key role in the perception that citizens have of them, which implies that policy makers should consider them in the construction of their messages in the different media where they appear.

In various investigations the influence of the body language of politicians as a source of persuasion has been analyzed and, more precisely, facial expressions, by traditional techniques like surveys (Stewart et al., 2009), with the use of eye tracking techniques (Gong and Bucy, 2016), with facial expression analysis through automatic detection systems (see e.g., McDuff et al., 2013; D'Errico and Poggi, 2019) and even with automatic speech recognition (Gupta et al., 2018). Also, information transmitted by TV can shape what individuals take in, influence their perceptions, convictions, and views regarding prevailing events and issues, and convey knowledge and interpretation (Mihăilă and Branişte, 2021). The interest about facial recognition and emotion sensing technologies is demonstrated by its application in various environments like predictive policing to optimize the police performance (Bacalu, 2021) and in the development of reality-based body enhancement technologies aimed at the algorithmic evaluation of body image and specifically the perception of the face (Lăzăroiu et al., 2017).

Understanding the influence of facial expressions in the communication of political leaders is fundamental for different reasons, as Gong and Bucy (2016) argued. Firstly, televised debates are the moment in which the messages of the candidates have greater coverage and impact on television audiences. Audience rates are very high, concentrating millions of viewers. Second, viewers appreciate not only the arguments, but also perceive and react to non-verbal language, in which facial expression is a key factor. The facial expressions of politicians in their interventions are a reflection of the emotions they experience at all times and influence the spectators. Third, the features of non-verbal communication are predictors of the viability and success of candidates.

The discussion about how the facial expression of emotion affects other people like the audience of a public debate, supported by the theory of mirror neurones, proposed by Rizzolatti and Sinigaglia (2008). This theory states that a certain type of neurones is activated when people not only perform an action, but also when those same actions are performed by other people, which leads to mimic their feelings and get their mood (see Carr et al., 2003). Mirror neurones are related to imitation or empathy and can interpret other people's facial micro expressions and understand how they really feel. Gestures and facial expressions can be controlled by repeated trials, but they can also occur outside the person's control. In any case, whether under deliberate control or outside it, the mechanism of mirror neurons allows people in the audience to understand and identify with the emotions of the candidate (Carr et al., 2003).

One of the most interesting components in the communication of political leaders is their facial expressions. These are the reflection of the emotions that a person experiences at all times, since it has been proven that these emotions have associated a series of motor responses that are observable in facial expressions (see Ekman, 1992). When experiencing an emotion, a reflex reaction is triggered, automatically, in the expression of the face (Redorta et al., 2006), since there is a brain structure that transmits the cerebral impulses from the processing centers of the emotions to the muscles of the face, giving rise to facial expressions (see Fernández-Abascal et al., 2010). Not all facial expressions are visible to the naked eye. A series of them, the micro expressions, those that have their origin in the subcortical layers of the brain and that are outside the voluntary control of the person, are the most difficult to detect, since their duration ranges between 1/25 and 1/5 s (see Ekman and Friesen, 1969).

**Figure 1:**
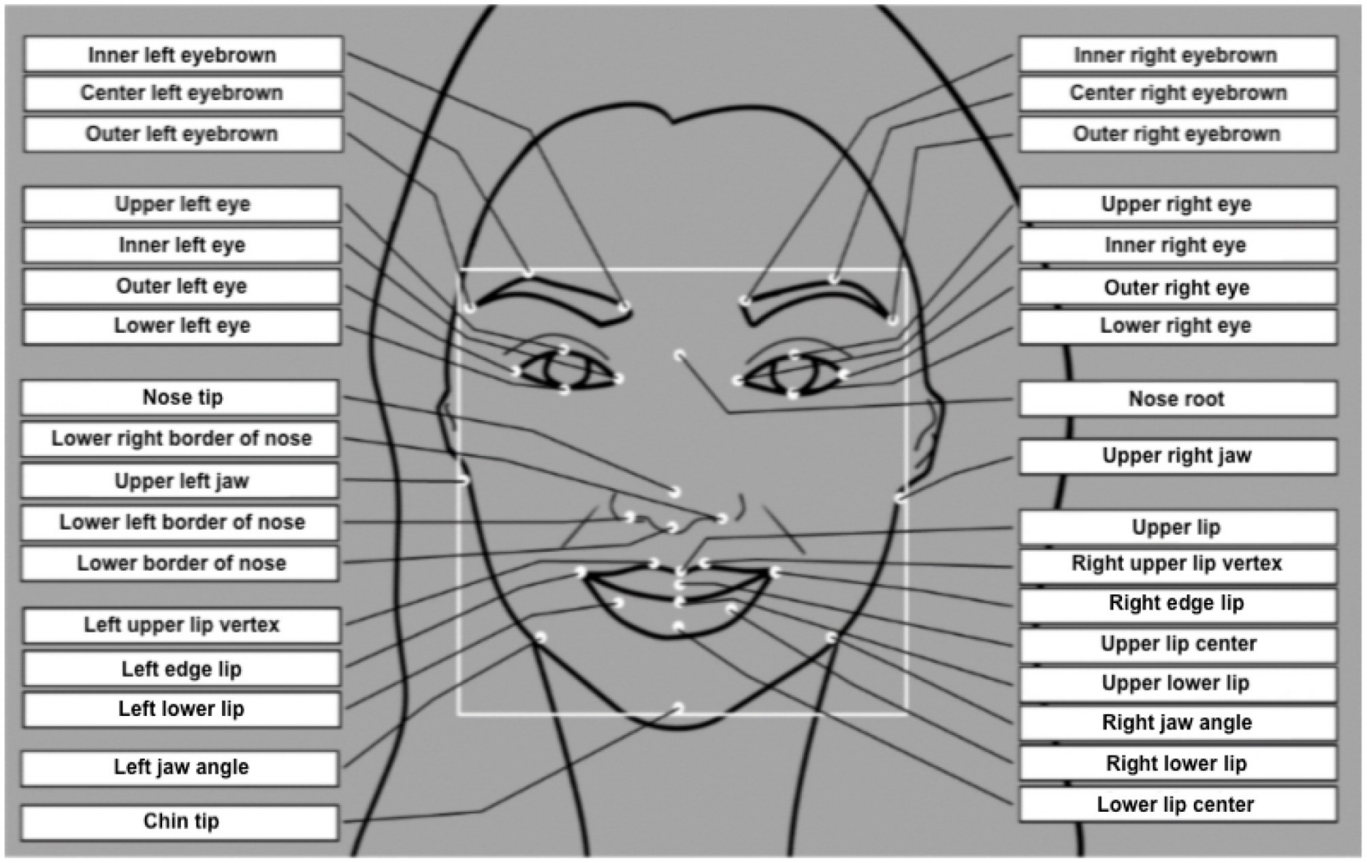
The different movements of human facial muscles as predictors of emotions based on the Facial Expressions Analysis (FEA) and Facial Action Coding System (FACS).

Facial Expressions Analysis (FEA) is one of the main techniques used in neuromarketing research as stated by Fortunato et al. (2014). This theory was developed by Ekman and Friesen (1978) as a way to identify the basic emotions by analyzing facial expressions. They proposed to identify facial expressions based on the combination of 46 basic muscle movements called Action Units (AUs) (see Figure 1). These AUs are the smallest movements of the muscles of the face that can be visually captured by a person. Each emotion is presented as a combination of several AUs. Ekman (1992) identified six basic emotions that are observable in people's facial expressions: anger, disgust, fear, joy, sadness, and surprise. Subsequently, contempt emotion was also included in this relationship (see Ekman et al., 2013). These are universal and innate expressions of biological origin that work without involving the individual's consciousness and have a series of specific facial expressions associated with them (Durán et al., 2017; see Table 1) which includes the considered AUs for each basic emotion and also for the engagement and valence.

**Table 1:** Emotions with corresponding Actions units and Facial Action Coding System (FACS) descriptions adapted from Krosschell (2020).

**Emotion**	**Action units**	**FACS descriptions**
Joy	6, 12	Cheek raiser, lip corner puller
Surprise	1, 2, 5, 26	Inner brow raiser, outer brow raiser, upper lid raiser, jaw drop
Anger	4, 5, 7, 23	Brow lowerer, upper lid raiser, lid tightener, lip tightener
Sadness	1, 14, 15	Inner brow raiser, brow lowerer, lip corner depressor
Disgust	9, 15, 16	Nose wrinkler, lip corner depressor, lower lip depressor
Fear	1, 2, 4, 5, 7, 20, 26	Inner brow raiser, outer brow raiser, brow lowever, upper lid raiser, lid tightener, lip stretcher, jaw drop
Contempt	12, 14	Lip Corner Puller, Dimpler
Engagement	1, 2, 4, 6, 9, 12,	Inner brow raiser, outer brow raiser, brow lowerer, cheek raiser, nose wrinkler, lip corner puller,
	15, 17, 18, 24, 25, 28	lip corner depressor, chin raiser, lip puckerer, lip pressor, lips part, lip suck
Valence	1, 2, 4, 9, 10, 12,	Inner brow raiser, outer brow raiser, cheek raiser, nose wrinkler, upper lip raiser, lip corner puller,
	13, 17, 24, 28	cheek puffer, chin raiser, lip pressor, lip suck

## 3. Materials and Methods

In this section we are going to explain how to measure the emotions of political candidates during election debates. Particularly, this article has focused on the analysis about the facial expressions of the main Spanish politicians considering the televised debate in the last general elections held in Spain on April 28, 2019. This debate took place on April 22 in the public network RTVE and the signal was broadcast in 11 chains. We have downloaded the video from the following source https://www.rtve.es/alacarta/videos/especiales-informativos/especial-informativo-debate-cuatro/5159816/. The four candidates who represented the greatest intention to vote were Pedro Sánchez (PSOE), Pablo Casado (PP), Albert Rivera (Cs), and Pablo Iglesias (UP). We denote the acronym of the political party they represent in parentheses. In the last section of this debate each participant had a space of one minute to launch their final plea to the voters. This phase is known as the golden minute, since each candidate generally summarizes their proposals, emphasizing the most important content. At this time, the audience exceeded 9.2 million viewers (see https://www.barloventocomunicacion.es/informes-barlovento/debate-electoral-22-abril-2019/).

During this short space they must use the best strategy in both content and form to capture attention and persuade. The management of verbal and non-verbal communication, as well as the internal structure of the message, its coherence, credibility, rhythm of the speech, are essential to reach viewers and get their message be remembered and achieve the intended objectives. For those reasons, candidates usually prepare this golden minute in detail and with their teams of communication advisors. This is the main justification for which this paper has been focused on the study of this particular time, following the ideas proposed by López-López et al. (2020). In this sense, it is important to note that one minute is the maximum time they have, although not everyone uses it exactly (i.e., the second candidate uses only 42 s). The length of their speech does not affect the analysis since the analysis exclusively considers the percentage of emotions in that period.

Nevertheless, although the timing of the video clip is different, the activation of the emotion is immediate as shown by different neurological studies. In this sense, the research proposed by Fino et al. (2019) point out that, seeing and reading about someone's smiling or frowning automatically elicits Emotionally Congruent Facial Responses (ECFR) in the observer or reader and this effect appears largely unconscious and difficult to suppress. For instance, within 500 ms after seeing a smiling face there is an activation of the zygomaticus major, the muscle that lifts up the corners of the mouth forming a smile. It is intended to associate the emotions expressed by the candidates with moments in their speech and not so much to measure coherence quantitatively. As Gillis and Nilsen (2017) note, listeners are exposed to inconsistencies in communication; for example, when speakers' words (i.e., verbal) are discrepant with their demonstrated emotions (i.e., non-verbal). Such inconsistencies introduce ambiguity, which may render a speaker to be a less credible source of information. More specifically, Beattie (2016) states that the coherence of non-verbal delivery with verbal statements likely affects the credibility and appropriateness of candidate statements.

We are going to apply a facial expression technique to measure the emotions that those politicians express and transmit during their speech. Particularly, we are going to deal with the seven basic emotions (joy, anger, fear, surprise, disgust, sadness, and contempt) defined by Ekman and Friesen (1978). We will also consider two aggregate indicators called valence and engagement (see Table 1). Let us remark a brief description about the variables we have taken into account in this article.

1. Joy is a pleasant feeling that produces pleasure. Choliz (2005) refers to it as a pleasant, desirable state that generates a feeling of well-being. Joy produces positive attitudes toward oneself and others, and favors the reception and positive interpretation of environmental stimuli. In psychology, joy is considered the most intense manifestation of happiness. Traditionally, joy has been associated with a positive valence, although it is possible as stated by Bowen (2016) that in some cases an expression of joy can happen with a negative valence.
2. Anger is an unpleasant sensation, which works as a hostile defense reaction against fear, pain, or a threat. A person feels angry when perceiving that he/she or someone who cares has felt offended, when he/she is sure of the nature and cause of the enraging event, when he/she is convinced that someone else is responsible and when he/she feels that they can still influence in the situation or deal with it (Choliz, 2005). In the same sense, and following this same emotional point of view, Videbeck and Videbeck (2013) points out that anger works as a defense mechanism against fear, pain, or sadness, assuming an intense hostile reaction to provocation, damage, or threat. In political communication, this expression is interpreted as a sign of seriousness and irritation that allows to emphasize the message, which is key from the political point of view (see D'Errico and Poggi, 2019).
3. Fear reflects a situation of anguish at the threat of possible harm. It is an emotion that acts as a defense mechanism against a potentially dangerous situation (Choliz, 2005). It is one of the most intense and unpleasant emotions, which provides apprehension, restlessness and discomfort. The manifestation of this emotion in the face of a politician could send a little positive message to the audience and cause some kind of cognitive dissonance on the part of the receivers (see Crawford, 2000).
4. Surprise appears as an emotional reaction to an unforeseen stimulus and which, according to Reeve et al. (1994) disappears quickly to give way to other emotions consistent with that stimulus. It is therefore, a short-lived transitional state that gives way to other subsequent emotional reaction. While the rest of emotions are associated with a positive or negative valence, surprise is considered a neutral emotion, which can have an ambivalent character and which can manifest itself with a positive or negative valence.
5. Disgust is an unpleasant impression that appears as a result of an awkward situation caused by an accident or disappointment. It also arises as a reaction to offensive stimuli of bad taste or unpleasant. According to Choliz (2005), disgust is emotion where physiological reactions are more evident. Expressions of disgust have a biological origin; hence they may appear to physical sensations as unpleasant gastrointestinal symptoms. Because this emotion generates a response of avoidance or withdrawal response should not be the most appropriate expression to show in the message during of the last television minute of the debate.
6. Sadness is related to affliction, grief or melancholy. It appears in situations of discouragement, melancholy or depression, and is associated with a loss of energy. According to Sternbach (1982), sadness reflects a situation of disappointment. It appears in situations of helplessness, lack of prediction, and control. These subjective experiences suggest that this emotion would not be the most appropriate expression that a political candidate should consciously reflect when presenting their messages to the audience. However, as noted by Ekman (1992), because these expressions occur outside the conscious control of the individual, the candidate may be unable to manage this negative reaction.
7. Contempt is an emotion that is linked to disrespect or lack of recognition. It is defined as an emotional reaction toward an individual or target group that is perceived as morally or socially inferior to oneself (Zhou, 2011). In this same line, Izard (2013) shows that the contempt reflects the superiority of one person compared to another.
8. Valence is an indicator of the positivity reflected in a person's face. If the state is of well-being, pleasant, the valence is positive, while, if there is a state of displeasure or discomfort, the valence is negative. Of the seven basic emotions, one of them has a positive character, joy, another has a neutral character, surprise, and the remaining five emotions have a negative character. The valence ranges on a scale from −100 (more negative) to +100 (more positive).
9. Engagement is synonymous with commitment, enthusiasm, intensity, or emotional involvement. It is obtained by analyzing the activation of the muscles of the face and reflects the expressiveness of the individual. According to Schaufeli et al. (2002), engagement refers to a positive mental state characterized by vigor, dedication, and absorption. In communication it is considered positive to show a high level of engagement to be effective in the emitter's objectives (see Heath et al., 2018).

In this sense, there are basically two automated methods for collecting data about the described emotions. The first one is the facial electromyography activity, for a review see e.g., Schulte-Mecklenbeck et al. (2017) and Wolf (2015). In this article, we will focus on the second one, the computer-based video classification algorithms because allow us to automatically encode the 46 facial expressions considered as well as to identify the emotions we have just identified.

Nowadays there are three main software environments which implement those classification algorithms. Noldus's FaceReader proposed by Van Kuilenburg et al. (2005), iMotions's AFFDEX module developed by McDuff et al. (2014), Zeng et al. (2008), El Kaliouby and Robinson (2005), and iMotions's FACET module provided by Littlewort et al. (2011). Particularly, we have considered the iMotions's AFFDEX platform because it has been scientifically validated in different studies in a context similar to ours (see e.g., Stöckli et al., 2018; D'Errico and Poggi, 2019; Kulke et al., 2020). We have also considered the classification algorithms given by the iMotions's AFFDEX module because the micro expressions can be analyzed and observed and related to the moment of the candidate's speech. In turn, the intention of the sender could be analyzed and the verbal message associated with facial expression through those machine learning tools. Let us explain how we have done it.

**Figure 2:**
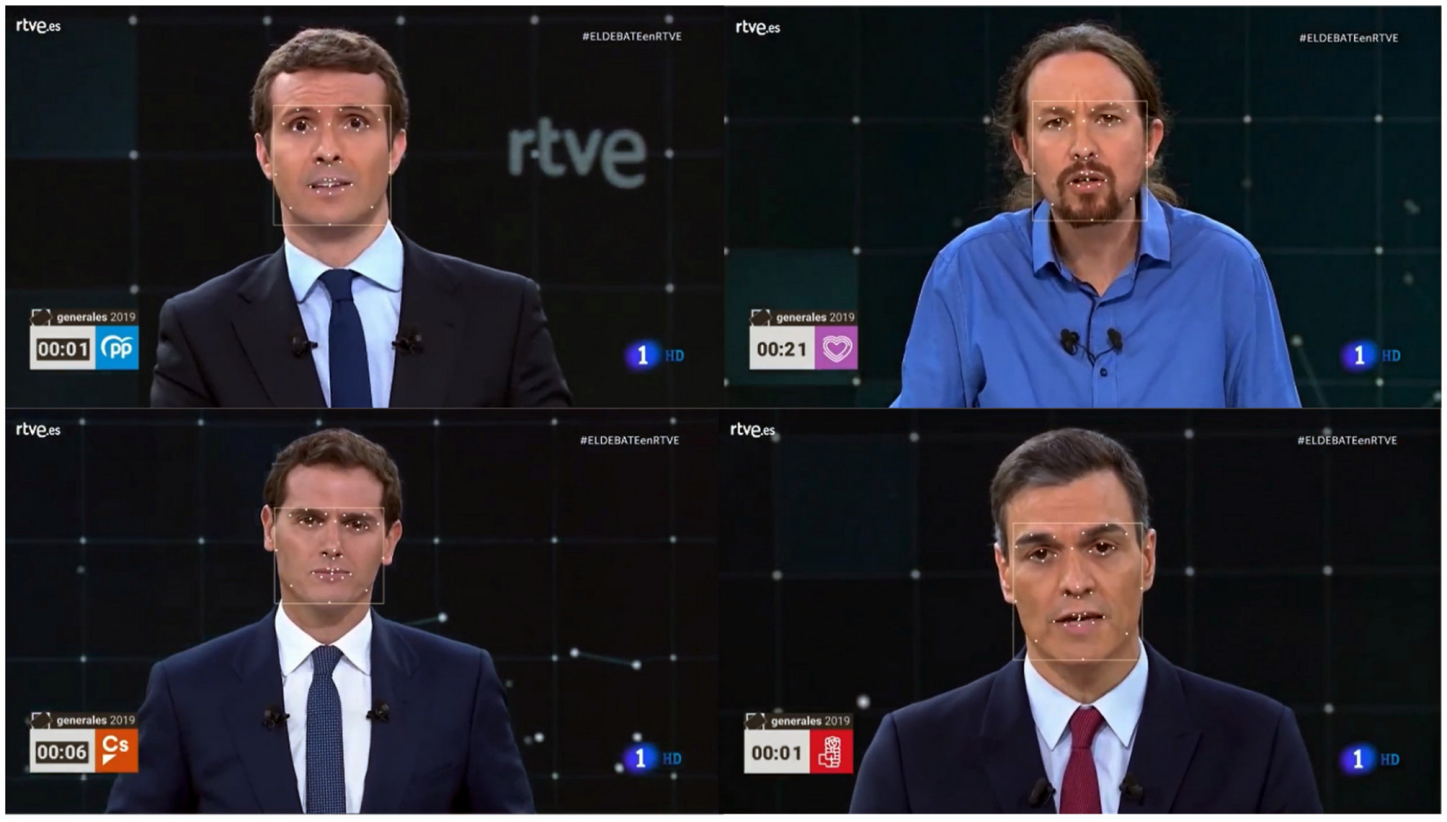
Application of Facial Expression Analysis on the four candidates using Affdex technology.

**Figure 3:**
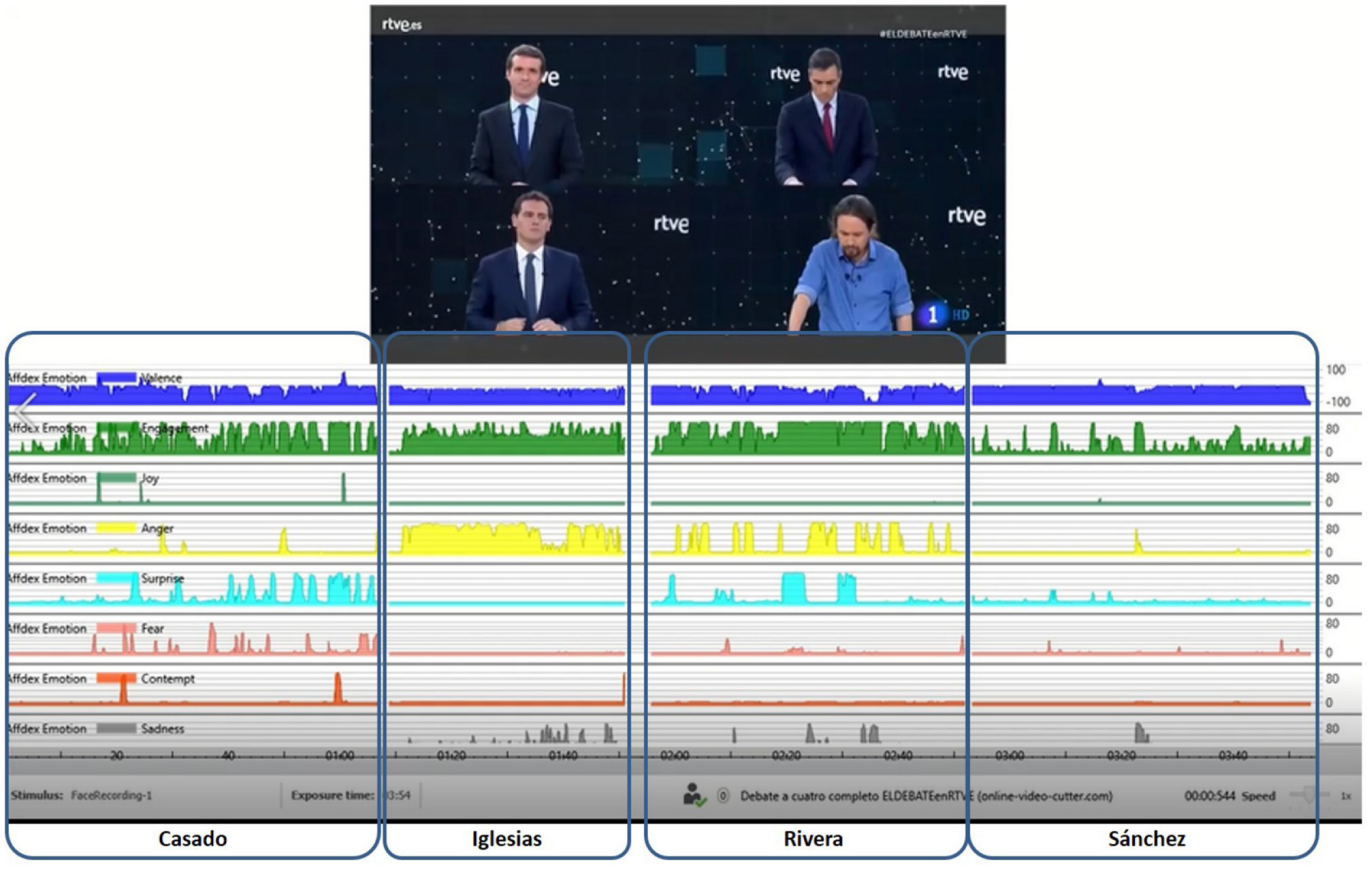
Graphical evolution about the emotions considered along the golden minute by each candidate. The blue shaded column corresponds to the time period analyzed for every candidate. Each row corresponds to the different emotions considered (in order): valence, engagement, joy, anger, surprise, fear, contempt, and sadness.

The video with the final interventions of each candidate (golden minute) has been post-processed through the AFFDEX module integrated in the iMotions platform. In this case, we have considered a total of 5.509 frames based on the four candidates corresponding to the final minute of exposure. That is, 25 frames per second have been analyzed. The application AFFDEX have identified on each frame the 46 facial expressions considered as well as the emotions described before (see Figures 2, 3). Having explained how we have obtained these data, let us focus on how we have analyzed them.

## 4. Results

In this section, we report the main empirical results of this paper. Particularly, we are going to study the communication of Spanish politicians during their interventions in election debates considering the information we have previously collected. This dataset is available on request to the corresponding author. Firstly, we have identified the emotions that each candidate shows during that time period and we have made a comparison between the four candidates considering seven basic emotions and two aggregate indicators.

Figure 3 shows the different evolution of each candidate by several emotional indicators during their golden minute: Valence, Engagement, Joy, Anger, Surprise, Fear, Contempt, and Sadness. As one can see, the four politicians reflect in their face more negative emotions than positive, although the emotions that appear throughout each speech are different, reflecting different emotional states, which are perceived by the audience and can influence the perception and assessment of each candidate. In this sense, anger appears most frequently in the interventions of Iglesias and Rivera (3rd and 4th candidates according to voting intention surveys). It can be interpreted as a sign of seriousness and irritation that aims to emphasize their message, as D'Errico and Poggi (2019) point out. In order of appearance, in Casado the two emotions that appear most frequently are surprise and fear; in Iglesias, it is anger; and in Rivera, it is also anger (although to a lesser extent than Iglesias) and surprise. In Sánchez, emotional activity is minimal.

**Figure 4:**
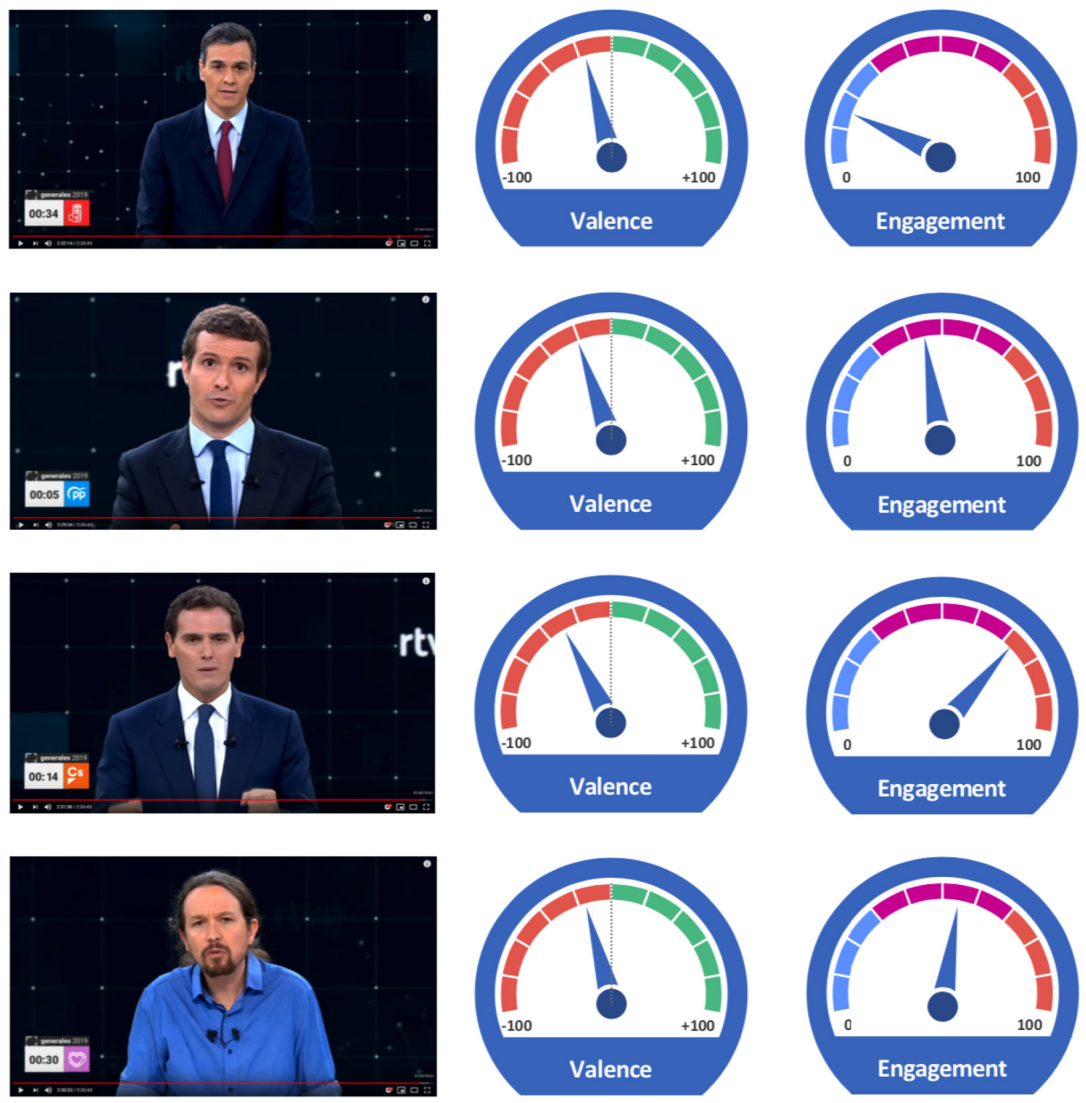
Comparison of the average values of valence and engagement shown by each candidate during the period analyzed.

Regarding the valence of emotions (see Figure 4), it is observed that at all times this indicator moves in negative zones, although it must be specified that for each participating candidate the negative values have different origins in each case. This different variation may come from the predominance of one or more negative emotions. The predominance of negative emotions in public interventions by politicians has been studied by D'Errico and Poggi (2019) justifying that negative valence as a means to reinforce the importance of the issues or topics that politicians talk about in every moment of his intervention. As for valence, Rivera is the candidate who has a more negative average value than the rest of the participants. On the other hand, the other three candidates maintain similar levels in this indicator, although always within a negative range. As for engagement, the highest value corresponds again to the Rivera candidate. In this sense, it is observed that in his intervention he makes a more intense use of body language, making greater emphasis on each part of his intervention. The average neutral value of engagement corresponds to Sánchez who had a more conservative and less expressive behavior during his intervention.

Then three types of behavior can be seen in terms of facial expressions observed and that are identified in the positions within the figure. In this sense, one type would correspond to the candidate Rivera (Cs) with a high engagement data and the most negative value of Valence. On the other end would be the candidate Sánchez (PSOE) with the minimum engagement value and the least negative of the Valence indicator. The other two candidates, Iglesias (UP) and Casado (PP), show average levels of the indicators analyzed. As for the raw data provided by the iMotions platform on emotions, they were calculated to obtain a binary result with a threshold of 10, meaning that all those facial expressions that, with at least 10% probability, were rated as such by a human evaluator are given as valid (see Table 2). This criterion is the one used by e.g., Kjærstad et al. (2020) and Stanley and Webster (2019).

**Table 2:** Descriptive analysis about the timestamp and frequency of the emotions showed by each candidate.

	**Casado (PP)**	**Iglesias (UP)**	**Rivera (Cs)**	**Sánchez (PSOE)**
Time (golden minute)	61.9 s	42.1 s	55.9 s	60.4 s
Timestamp	1,547 frames	1,052 frames	1,399 frames	1,511 frames
No. Emotions	813 emotions	1,623 emotions	1,000 emotions	165 emotions
No. Emotions/Timestamp	53% frames	154% frames	72% frames	11% frames
Variable 1: **Joy** (times-%)	23 (2.8%)	–	–	1 (0.6%)
Variable 2: **Anger** (times-%)	74 (9.1%)	836 (51.5%)	494 (49.4%)	26 (15.8%)
Variable 3: **Surprise** (times-%)	467 (57.4%)	–	279 (27.9%)	47 (28.5%)
Variable 4: **Fear** (times-%)	184 (22.6%)	–	40 (4.0%)	14 (8.5%)
Variable 5: **Contempt** (times-%)	43 (5.3%)	6 (0.4%)	–	–
Variable 6: **Sadness** (times-%)	14 (1.7%)	772 (47.6%)	141 (14.1%)	76 (46.1%)
Variable 7: **Disgust** (times-%)	8 (1.0%)	9 (0.6%)	46 (4.6%)	1 (0.6%)

The shortest intervention corresponds to the second candidate in order of appearance, Iglesias, who only used 42 of 60 s available, while the rest of the candidates fit the time available. In two of the candidates, Casado and Rivera, the emotion surprise has a wide presence (57.4 and 27.9% of the frames, respectively). The emotion surprise is considered as a quick and instantaneous emotional reaction that gives way to other emotions (see Reeve et al., 1994). In Casado's case, the emotion that usually appears next is fear, while in Rivera's case it is anger. In the case of candidate Sánchez, the base of frames with emotions is so low that its analysis is meaningless.

The candidate Iglesias, despite being the one who spends the least time in his speech, is the one who shows the most emotional activity, with an average of 1.54 emotions per frame, well above the other candidates (Rivera: 0.72; Casado: 0, 53, and Sánchez: 0.11). Now we are going to associate basic emotions with specific moments of the candidate's speech, identifying the topics they address and relating them directly to the expressed emotion. In this case, we want to identify, through a qualitative approach, the main phrases in the speech of every politician during the golden minute associated with certain emotions. Then we are going to check whether the differences shown by each candidate are statistically significant or not. Results from the analysis about the four candidates, in order of appearance in the golden minute, are shown below.

**Figure 5:**
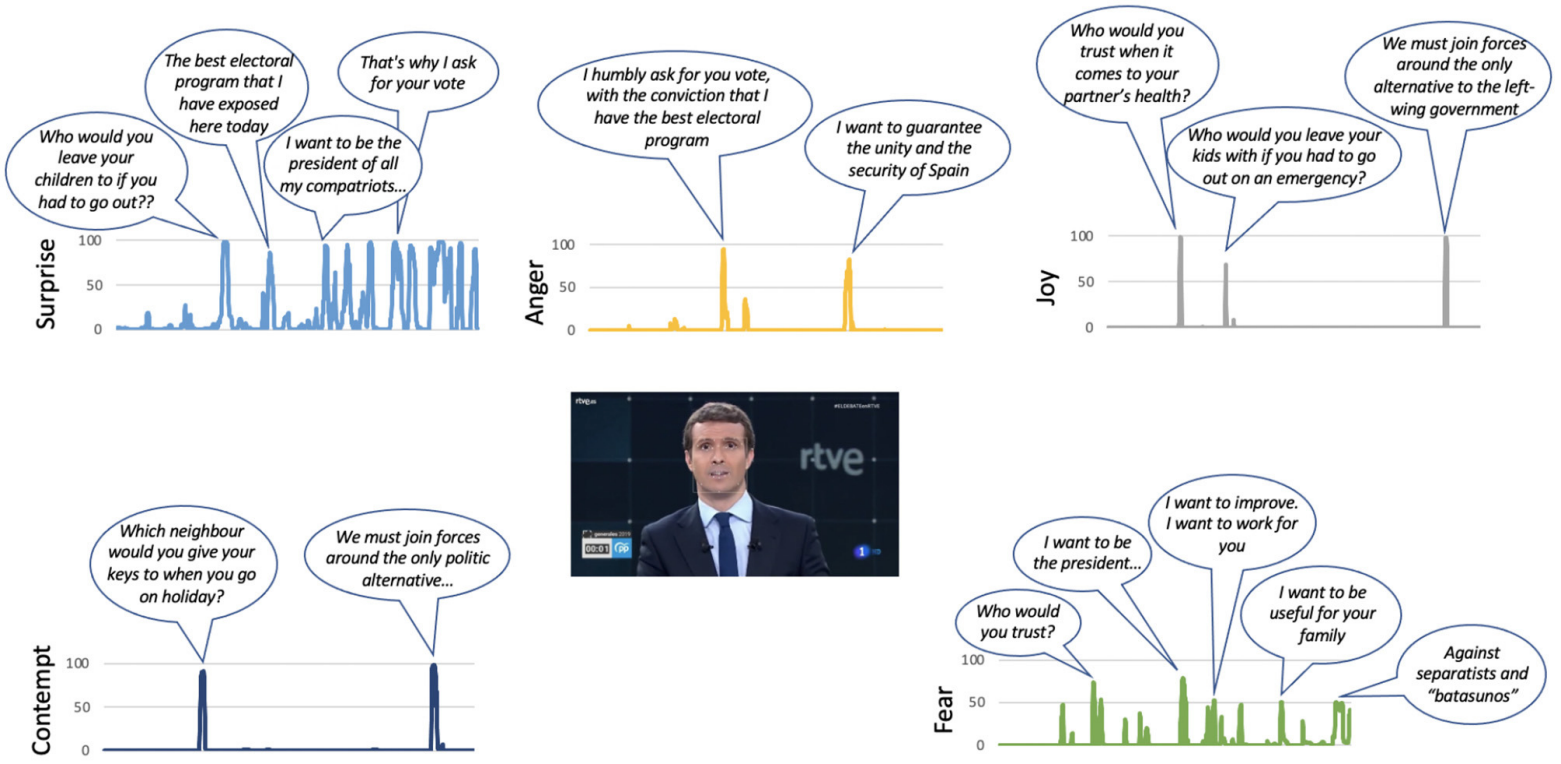
The main phrases of Pablo Casado's speech during the golden minute associated with certain emotions.

**Figure 6:**
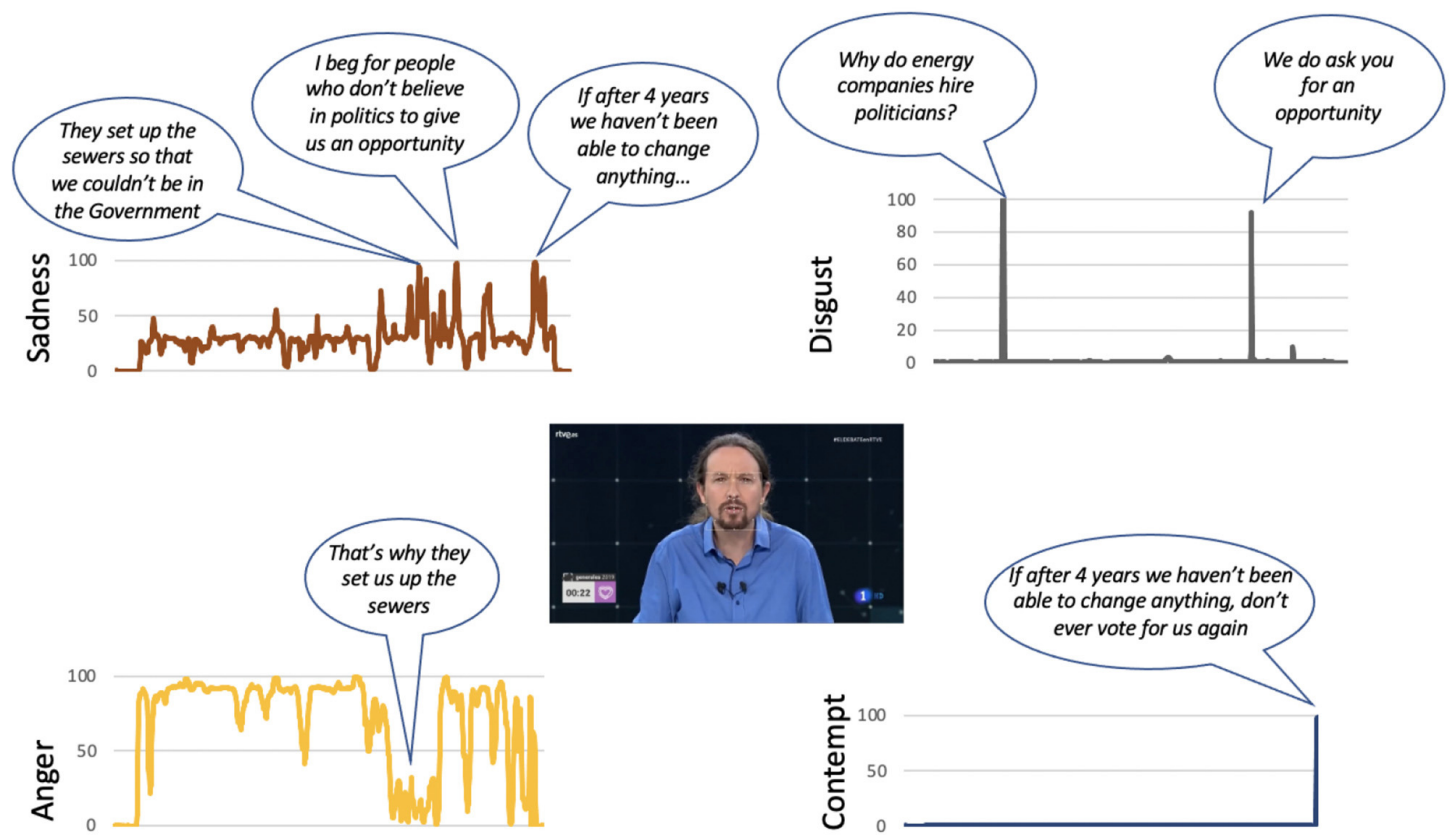
The main phrases of Pablo Iglesias's speech during the golden minute associated with certain emotions.

**Figure 7:**
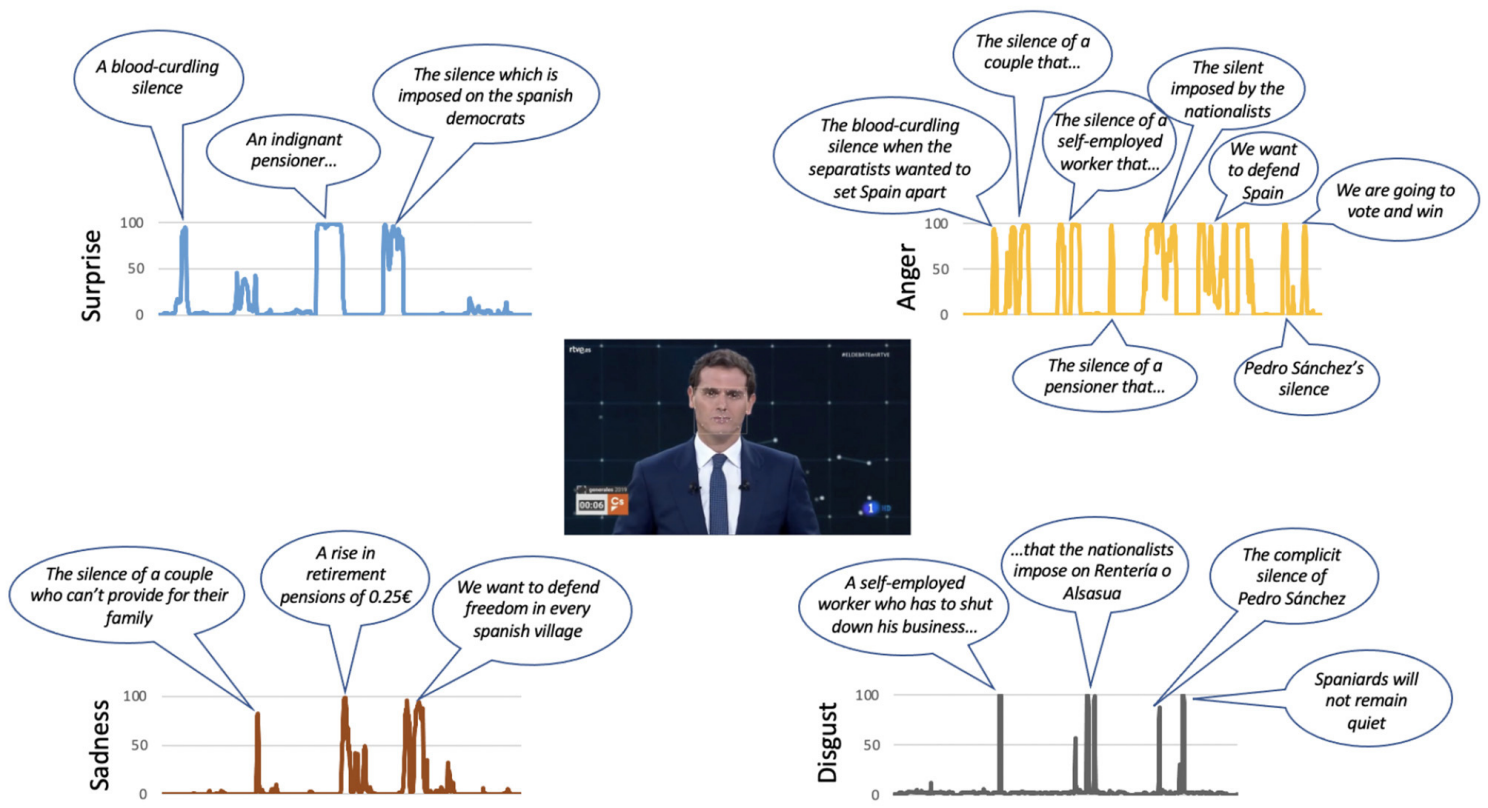
The main phrases of Albert Rivera's speech during the golden minute associated with certain emotions.

**Figure 8:**
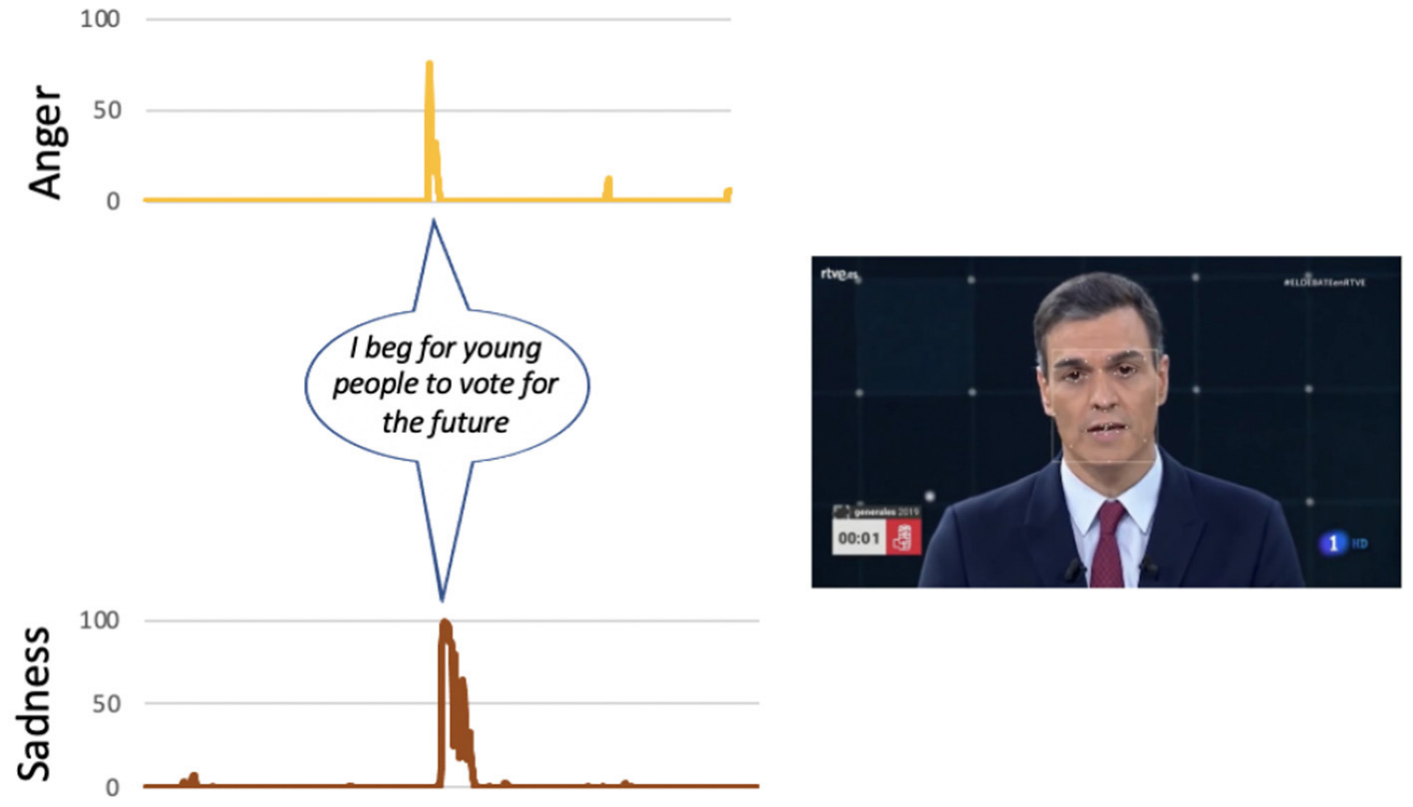
The main phrases of Albert Rivera's speech during the golden minute associated with certain emotions.

- Casado (PP): the two emotions that are most active in his speech are surprise and fear that appear simultaneously on some occasions. At no time is it observed that anger appears. The emotion surprise appears as his speech progresses, associated with the moments in which he expresses who will be the president (“I want to be the president of all my compatriots: those who vote for me and those who don't, those who insult or support us …,” “I want to work for you and serve Spain,” or “we have to join forces around the only alternative on the left”). The presence of fear in some moments of his intervention seems to diminish the content of his message. This emotion is observed when making statements such as “I want to be the president,” “I want to work for you,” or “we have to join efforts around the only alternative…” (see Figure 5).
- Iglesias (UP): his intervention presents a greater coherence between the emotions identified in the facial expressions analysis and the content of his speech. The two emotions with the highest values during his intervention are anger and sadness. The emotion that appears constantly in his intervention is anger. This emotion only disappears when the candidate refers to the attacks suffered by his party (“and that's why we seized the sewers”). The sadness appears in moments of the speech in which it is presented in a victimistic role (“nobody buys us,” “prevented us from entering the government”). It also appears at the final moment when he says “if in four years we have not managed to change anything, do not vote for us anymore.” There is also a negative emotion that appears in the last sentence of his speech, which is contempt, when he reaffirms a message he had already said: “If after those 4 years we have not managed to change anything ...” (see Figure 6).
- Rivera (Cs): it is the participant who makes greater use of rhetorical resources during his intervention. He uses the question “do you hear it?” at different times along the golden minute. It is observed that every time he raises this question, anger arises to reinforce the message he expresses below. The sadness appears when referring to certain disadvantaged groups (“they cannot have children...,” “their pension has risen only 0.25 euros”), and by appealing to freedom (“we want to defend our freedom in any town in Spain”; see Figure 7).
- Sánchez (PSOE): he is the candidate who least conveys emotions in his intervention, probably guided by a more conservative strategy, since at that time he was president and the polls gave him the winner of the elections. In three of the seven emotions analyzed, he is the one that shows the minimum values, specifically the emotions of anger, disgust, and contempt. His intervention is the flattest of all candidates. There is only one expression of sadness when addressing young people (“I ask young people to vote for the future”; see Figure 8).

Once the emotions have been identified with certain moments of the discourse, we are going to carry out the following hypothesis tests. On the one hand, we want to know whether the differences shown by each candidate in every emotion are statistically significant. We will do this by applying the non-parametric chi-squared goodness-of-fit test. On the other hand, by applying the ANOVA analysis we will contrast whether, on average, there are differences between the candidates considered. Let us start with the first test.

In this case, the purpose of the chi-square test is to compare the possible differences between the observed frequencies for each emotion by taking the results shown in Table 2, for a review of this technique applied in this context (see e.g., Mancini et al., 2020; Sei and Ohsuga, 2021). We are going to calculate the chi-squared statistic denoted by χ^2^ and test the following null hypothesis *H*_0_:*f*_*i*_ ≠ *f*_*j*_∀*i* ≠ *j* where *f*_*i*_, *f*_*j*_ report the observed frequencies for different emotions and *i, j* = joy, anger, fear, surprise, disgust, sadness, and contempt. Note that we have to carry out this test for each candidate individually. To do so, we will use the function chisq.test from the library called stats which belongs to the R open-source programming language. This algorithm is publicly available at CRAN repository https://www.rdocumentation.org/packages/stats/versions/3.6.2/topics/chisq.test. Results are shown in Table 3.

**Table 3:** Chi-squared test results considering the observed frequencies for each emotion showed by every candidate.

	**Casado (PP)**	**Iglesias (UP)**	**Rivera (Cs)**	**Sánchez (PSOE)**
Chi-square	7.361	8.272	6.934	7.157
*p*-value	0.769	0.793	0.712	0.759
Sign. value (α)	0.05	0.05	0.05	0.05
Decision rule	*p*-value > α	*p*-value > α	*p*-value > α	*p*-value > α
Result *H*_0_	Not reject *H*_0_	Not reject *H*_0_	Not reject *H*_0_	Not reject *H*_0_

*The null hypothesis is H_0_ : f_i_ ≠ f_j_∀i ≠ j where f_i_, f_j_ report the observed frequencies for different emotions from Table 2 and i, j = anger, disgust, fear, joy, sadness, surprise, and contempt*.

For a significance level equal to 5% we can see how we obtain higher p-values for all candidates. Then, applying the decision rule, we do not reject the null hypothesis *H*_0_:*f*_*i*_ ≠ *f*_*j*_∀*i* ≠ *j*. Therefore, we can affirm that the differences between the observed frequencies for each emotion by taking the results shown in the Table 2 are statistically significant for all candidates. Now we are going to check whether, on average, there are differences between the candidates considered. In this case, we will use the 1-factor ANOVA test, for a review of this technique applied in this context (see e.g., Goretzko et al., 2019; Wang and Deng, 2021). To carry out a 1-factor ANOVA analysis, we must have one response variable (dependent variable that we want to explain) and one factor (independent variable that we use to explain) with two or more levels (values of the independent variable). In this sense, we want to study whether there are significant differences between the levels of the indicators (on average) about the seven basic emotions that are observable in people's facial expressions: joy, anger, fear, surprise, disgust, sadness, contempt. We consider the type of politician as a factor. Defining the following levels of this factor: Casado (PP), Iglesias (UP), Rivera (Cs), Sánchez (PSOE).

The null hypothesis states that all means of the factor levels are equal while the alternative hypothesis states that at least one is different. That is, *H*_0_:μ_1_ = μ_2_ =... = μ_*i*_ with *H*_1_:μ_*i*_ ≠ μ_*j*_ for some *i, j* where *i, j* = Casado (PP), Iglesias (UP), Rivera (Cs), Sánchez (PSOE), and μ_*i*_ is the average indicator of the seven basic emotions showed by the candidate *i*. In this case, we do not consider the variables valence and engagement as they are two aggregate indicators whose interpretation is not comparable to the case of basic emotions. Our objective at this point would be to obtain numerical evidences about our research question by calculating the mean statistic for each factor level. Then, we have to make these numerical evidences robust by applying the hypothesis test provided by the ANOVA analysis using the aforementioned null hypothesis. To do so, we will use the aov function of the library called stats which belongs to the R open-source programming language as well. This algorithm is publicly available at CRAN repository https://www.rdocumentation.org/packages/stats/versions/3.6.2/topics/aov. The results obtained are shown in Table 4 and contains the following information (by columns):

- Name of the factor, in this case, candidate or politician, and residuals, which represents the errors or residuals from applying the ANOVA analysis (the error term).
- Degrees of freedom, labeled Df, gives the degrees of freedom corresponding to the factor (its number of levels −1) and the residuals (the number of basic emotions multiplied by four candidates, minus the number of levels of the factor).
- Sum Sq, shows us the sums of the squares of the factor (SCE), and the residuals (SCD).
- Mean Sq, contains the means of the factor squares, (MCE = SCE/Df factor), and the residuals (MCD = SCD/Df error).
- *F*-value, gives the value of the contrast statistic used and it is equal to MCE/MCD.
- Pr(>F), gives the p-value of the hypothesis test used to find out whether or not we reject our null hypothesis.

**Table 4:** ANOVA analysis results provided by the four candidates.

	**Df**	**Sum Sq**	**Mean Sq**	***F*-value**	**Pr (>F)**
Basic emotions	3	228	76.0	16.52	4.66e-05
Residuals	24	112	4.6		

*The null hypothesis is H_0_ : μ_1_ = μ_2_ =... = μ_i_ with H_1_ : μ_i_ ≠ μ_j_ for some i, j where i, j = Casado (PP), Iglesias (UP), Rivera (Cs), Sánchez (PSOE) and μ_i_ is the average indicator of the seven basic emotions (joy, anger, fear, surprise, disgust, sadness, contempt) showed by the candidate i*.

For a significance level equal to 5% we can see how we obtain a lower p-value. Then, applying the decision rule, we reject the null hypothesis *H*_0_:μ_1_ = μ_2_ =... = μ_*i*_ where *i, j* = Casado (PP), Iglesias (UP), Rivera (Cs), Sánchez (PSOE), and μ_*i*_ is the average indicator of the seven basic emotions showed by the candidate *i*. Hence, we can say that the differences observed between the various candidates with respect to the basic emotions, on average, are statistically significant. That is, we have obtained statistical evidences that the means compared are not all equal.

Finally, let us show you the results provided by different surveys from the main media in Spain regarding the evaluation of the debate. Then, in the next section we will discuss whether there is consistency between these results and those obtained in our empirical analysis. After the debate was held on 22 April 2019, different media outlets conducted polls on their websites in order to find out the audience's opinion on who had won the debate. In total, 15 media outlets were identified and collected a total of 1,144,709 votes, the details of which are included in Table 5. The weighted average of all the votes shows Rivera as the winner of the debate (41.79% of the votes), followed by Iglesias (24.93%), Sánchez (18.81%), and Casado (13.80%). In the second column we have included the ideological profile of the press readers according to the results of the surveys based on the ideological positioning of the different media obtained from https://smreputationmetrics.wordpress.com/2015/12/09/el-perfil-ideologico-de-los-lectores-de-prensa-analisis-encuestas-7deldebatedecisivo/.

**Table 5:** Opinions about the winner of the debate considering the main media in Spain.

	**Media**	**Data collection date**	**Ideological position readers**	**N (obs.)**	***N* (%)**	**Rivera (%)**	**Casado (%)**	**Iglesias (%)**	**Sánchez (%)**
1	El Mundo	18/11/20	4.3	366.776	32	51.76	18.08	15.20	14.96
2	El País	23/04/19	4.0	221.511	19.4	38.51	8.92	26.86	22.20
3	Marca	18/11/20	–	101.538	8.9	39.00	13.00	29.00	19.00
4	Público	18/11/20	2.9	83.890	7.3	24.00	7.00	48.00	21.00
5	La Vanguardia	23/04/19	4.1	76.639	6.7	37.64	9.61	33.64	19.26
6	El Confidencial	18/11/20	4.5	70.202	6.1	49.00	17.00	18.00	16.00
7	El Español	23/04/19	4.5	63.235	5.5	50	23.00	14.00	13.00
8	La Voz de Galicia	18/11/20	4.4	37.220	3.3	32.00	12.00	30.00	26.00
9	Cope	23/04/19	–	32.862	2.9	42.00	21.00	27.00	10.00
10	Huffington Post	23/04/19	3.8	30.016	2.6	11.00	5.00	42.00	42.00
11	Europa Press	23/04/19	4.5	23.444	2.0	36.60	11	30.20	22.30
12	Heraldo	18/11/20	–	17.648	1.5	42.00	13.00	27.00	18.00
13	Cadena Ser	23/04/19	3.4	17.190	1.5	15.20	4.10	47.20	33.60
14	ABC	18/11/20	5.1	2.538	0.2	42.00	21.00	21.00	16.00
			**Total**	1.144.709	100				
				**Weighted mean**		41.79	13.80	24.93	18.81

The media whose readers are on the ideological right are Expansión, ABC, Onda Cero, and La Gaceta. Also further to the right than the average are El Confidencial and El Mundo. In the central zone are media such as Europa Press, El Español or 20 Minutos. Below the average, toward the center left, we find media such as El País, El Periódico, or El Huffington Post. One step below, in positions further to the left, are headers such as Cadena SER or Público.

## 5. Discussion

In an electoral campaign there are many and diverse public interventions carried out by candidates. Of all these interventions, televised debates are those that, in general, arouse the greatest interest from the audiences. The television channels themselves are what, through their advertising, promote, and encourage audiences to follow the debate. Within these debates, the so-called golden minute is a decisive space in which the participating candidates must design a message, both in their content aspects and in nonverbal aspects, that is highly persuasive. The goal is to deliver your proposal and convince voters, especially those who are undecided. Each candidate who participates in an electoral debate has a team of advisors that supports them in preparing this speech. However, facial expressions, especially micro-expressions lasting <1/5 of a second, which by their very nature are outside the person's conscious control, are aspects that are key and reveal the true emotional state of the individual.

In this sense, this article tries to add a practical value through the application of the facial expression analysis technique to the communicators themselves, which allows the identification and evaluation of the intensity of the expressed emotions. This application is a methodological contribution to the analysis of the communication of public figures. As already identified by D'Errico and Poggi (2019), the facial expressions shown by politicians at the most decisive moment of the debate (the golden minute) generally reflect negative emotions, as a way of reinforcing their arguments. However, it is observed that through the analysis of facial expressions, the emotions that condition the negative value of valence are different in each candidate. Thus, it is observed that, in the interventions of two of them (Iglesias and Rivera), a negative emotion frequently appears: anger, but it can transmit seriousness, transcendence, and confidence, while in the other two candidates this emotion is not observed.

Also differences were observed in the average indicators of valence and engagement of the four political leaders in their final intervention. This could reflect different communication styles and strategies. It was observed that the candidates who have a lower intention to vote at the date of the debate (Rivera and Casado), and therefore, those who have more need to connect with the electorate, are those who show the highest engagement rates. These are the cases of Rivera and Iglesias. On the contrary, the lowest engagement value corresponds to the candidate Sánchez, acting president at the time of the debate, and with greater intention to vote. Therefore, this may be the reason for assuming a more conservative communication strategy in the face of the risk of losing the confidence of both current and undecided voters. From the analysis of the results, it could be determined that there is no correlation between the emotion shown in the face and the verbal locution, at moments of the interventions of some of the analyzed candidates. This would be the case of the Casado candidate (PP) in which a dysfunction is observed when his intention to be president is alluded to, and the emotion that he expresses is surprise. This candidate also shows this lack of correlation between what he says and what his face expresses with the appearance of fear in some moments of his intervention. This lack of coherence could be interpreted as a loss of strength of the content of his message and especially in the credibility of the candidate.

On the other hand, the candidate Iglesias is where this dysfunction or lack of correlation between facial expression and verbal speech is not clearly observed in the analysis. That is why the appearance of a negative emotion is significant, such as contempt. It appears right in the final moment of his intervention, and that reflects superiority and arrogance over the rest, both his political opponents and the viewers. On the other hand, it is worth noting that this candidate is the only one who used the least time in relation to his opponent. In the speech of the candidate Iglesias, it is observed how an emotion that is present from the beginning of his speech, disappears at a certain moment to reappear later. It's about the emotion anger. Ekman (2009) states that deliberate expressions can be kept on the face with the intention of deceiving, but that involuntary expressions may appear at some point that are beyond the control of the person that can provide clues about a possible deception. It would be the case of the disappearance of the expression at that moment and that could indicate that the emotion anger that he wants to convey from the beginning is a false emotion. Regarding the candidate Rivera, coherence is observed when expressing the emotion of sadness when referring to certain disadvantaged groups, and also when it refers to concepts such as freedom. In the case of the candidate Sánchez, he is the one who transmits the least emotions in his intervention. Probably this more conservative position is conscious, since at that time he was already acting president and, in the polls, he appeared with greater intention to vote. According to the analysis data, it is observed that his intervention is the one that transmits the least emotionality. The results obtained have been statistically validated and robust evidence has been obtained in this respect.

## 6. Limitations

Concerning the limitations of this paper it is necessary to remark that the Facial Expression Analysis based on action units (AU) has been scientifically validated in different studies and contexts from an empirical point of view (see e.g., Stöckli et al., 2018; D'Errico and Poggi, 2019; Kulke et al., 2020; Otamendi and Sutil Martín, 2020). Behind this methodology there is a consolidated theoretical foundation and a whole research line as we have identified in the different papers cited in Section 2 (see e.g., Ekman, 1992; Carr et al., 2003; Fernández-Abascal et al., 2010; Fortunato et al., 2014; Durán et al., 2017). However, when measuring facial expressions there are a number of issues that need to be reconsidered in future research, such as the effect it could have the muscle movement caused by the speech, the viewing angle, the lighting and image quality, or even whether the person being analyzed has some kind of cosmetic or surgical operation on the face itself that may affect the analysis of facial expressions.

Also it is important to remark that it is not possible to have longitudinal datasets in this field considering the same politicians because the candidates usually change in each debate, as it is rare for the same politician to run for more than two elections. This fact does not invalidate this type of analysis at all, since we are not focusing on any specific politician, but rather on emphasizing that by choosing novel facial expression techniques to analyse the discourse of political leaders provides us with very interesting information when designing their communication policies. We can also identify the impact they can have on the electorate, the emotions they can transmit, and complements the rest of the classic analysis techniques considered in this context.

In this sense, we are aware that the results shown in this paper are scalable to other political debates occurring in Spain or in other countries as well as other types of public events of other personalities of interest. So we hope to see soon that studies similar to this one will appear considering in its analysis the computer-based video classification algorithms because allow us to automatically encode the facial expressions showed by the communicators as well as to identify their emotions. Another issue would be to check whether there is a correlation between the type of emotion projected by the politician and those generated in the audience. This line of work would allow us to deepen the application of the theory of mirror neurones put forward by Rizzolatti and Sinigaglia (2008). In the same way it is possible to extend the scope of this work by checking if the analysis of the emotions shown through the candidate's facial expressions has an effect on the public's assessment of who is the winner of the debate, and on the other hand in the own candidate assessment from the political position of each viewer. A study of this type has been carried out in the analysis of the campaign for the presidential elections of Peru held in June 2016 although focused on the rhetoric of the message (see Ortigueira-Sánchez and Cárdenas-Egúsquiza, 2019).

## 7. Conclusion

The communication of public figures in televised debates has been a frequent reason for research, using different approaches. A first line of analysis has highlighted the importance of the emotional component (see e.g., Crawford, 2000; McDuff et al., 2013; Ortigueira-Sánchez and Cárdenas-Egúsquiza, 2019; D'Adamo et al., 2021). A second line of research has confirmed non-verbal expression as a fundamental component in interpersonal communication, especially in political communicators (see e.g., Dumitrescu et al., 2015; D'Errico and Poggi, 2019). Finally, a third line has focused their analysis on discourse (see e.g., AlShehri, 2019). Aware of the value of the three factors (emotions, nonverbal message, and discourse) in communication, this paper has tried to analyze the association between the emotions expressed and the speech that is transmitted at each moment. This analysis allows access to new levels of analysis in the communication of public figures. Associating data from different sources, previously treated independently enriches the analysis. In this sense, this article provides a first step in trying to associate both analyzes: speech and facial expressions. That is, the simultaneous use of several techniques to deepen the study of the interventions can add a new dimension to the combined analysis of different types of data: those that come from the emotions expressed on their faces and the speeches expressed at those moments. The application of neuroscience techniques represents a very interesting and promising field of study in public communication, in which there are not yet too many solid research lines.

## Data Availability Statement

The raw data supporting the conclusions of this article will be made available by the authors, without undue reservation.

## Ethics Statement

Written informed consent was obtained from the individual(s) for the publication of any potentially identifiable images or data included in this article.

## Author Contributions

All authors have participated in all stages of work, including the conception and design of the research, the revision of intellectual content, and drafting the work. All authors contributed to the article and approved the submitted version.

## Conflict of Interest

The authors declare that the research was conducted in the absence of any commercial or financial relationships that could be construed as a potential conflict of interest.

## Publisher's Note

All claims expressed in this article are solely those of the authors and do not necessarily represent those of their affiliated organizations, or those of the publisher, the editors and the reviewers. Any product that may be evaluated in this article, or claim that may be made by its manufacturer, is not guaranteed or endorsed by the publisher.
